# Supplementary material for: ESGO/ESHRE/ESGE Guidelines for the fertility-sparing treatment of patients with endometrial carcinoma,
Source: Hum Reprod Open. 2023 Feb 6;2023(1):hoac057. doi: 10.1093/hropen/hoac057 (PMC9900425; doi:10.1093/hropen/hoac057)
Supplement: hoac057_Supplementary_Data1 [file hoac057_supplementary_data1.docx]

**Supplementary data 1. IDENTIFICATION OF SCIENTIFIC EVIDENCE**

| **Literature search in MEDLINE** | |
| --- | --- |
|  |  |
| Research period | 2016/09/01 - 2021/09/01 |
|  |  |
|  |  |
| Indexing terms | Abortion, adnexal pathology, adverse effect, adverse event, age, age factor, antimullerian hormone, anti-Mullerian hormone, antral follicle count, assisted reproduction, assisted reproductive technique, assisted reproductive technology, beta-catenin, beta-catenin testing, β-Catenin, β-Catenin testing, bilateral salpingo-oophorectomy, biomarker, biopsy, body mass index,chest imaging, chest radiology, clinical examination, clinical manifestation, clinical staging, colorectal neoplasms, comorbidity, completion surgery, complications, comprehensive surgical staging, comprehensive staging, computed tomography, conception, conservative therapy, conservative surgery, conservative treatment, CTNNB1, curettage, cytology, cytoreduction, cytoreductive surgery, definitive surgery, diagnosis, diagnostic performance, differential diagnosis, diffusion-weighted imaging, diffusion-weighted magnetic resonance imaging, dilation and curettage, disease, disease progression, early disease, early stage, ectopic, endometrial biopsy, endometrial cancer, endometrial carcinoma, endometrial hyperplasia, endometrial intraepithelial neoplasia, endometrial neoplasm, endometrial sampling, endometrioid endometrial cancer, estrogen, estrogen receptor, estrogen receptor status, experienced pathologist, experienced clinician, expertise, extrauterine disease, fertility, fertility outcome, fertility preservation, fertility-preserving treatment, fertility sparing, fertility sparing management, fertility sparing treatment, fertility sparing surgery, fertility status, fertilization *in vitro*, follow-up, follow-up protocols,gene mutation testing, gonadotropin-releasing hormone, gonadotropin-releasing hormone agonist, gross examination, health status, health-related quality of life, hereditary nonpolyposis, hereditary nonpolyposis colorectal cancer syndrome, histopathology, hormonal therapy, hormone therapy, human epidermal growth factor receptor 2, hysterectomy, hysteroscopy, hysteroscopic biopsy, hysteroscopic resection, hysteroscopy,imaging, immunohistochemical diagnosis, immunohistochemistry, *in vitro* fertilization,laparoendoscopic single-site approach, laparoscopic staging, laparoscopy, laparotomy, late recurrence, levonorgestrel intrauterine device, levonorgestrel intrauterine system, local control, lymphadenectomy, lymph node, lymph node assessment, lymph node dissection, lymph node involvement, lymph node staging, Lynch syndrome, Lynch syndrome patients, maintenance treatment, magnetic resonance imaging, management, marker, maximum standardized uptake value, medical treatment, medication, medroxyprogesterone, medroxyprogesterone acetate, megestrol acetate, metastasis, metastatic disease, metastatic tumour, metformin, microsatellite instability, mini-laparoscopic approach, mini-laparoscopic, mortality, mini-laparoscopy, minimally invasive approach, minimally invasive surgery,miscarriage, mismatch repair, molecular biology, molecular marker, molecular profile, molecular profiling, mortality rate, mortality analysis, multivariate analysis, myometrial invasion, myometrial involvement, myometrium,obese, obese patient, obesity, omentectomy, oncofertility, oncofertility consultation, oncofertility counseling, oral progestin therapy, oral progestogens, organ sparing treatment, ovarian preservation, ovarian reserve, overweight, p53, p53 mutated, p53 mutation, p53 testing, pathology, para-aortic lymph node, para-aortic lymphadenectomy, pathologist, pathology, patient selection, pelvic exenteration, pelvic lymph node, pelvic lymphadenectomy, percutaneous surgery, percutaneous surgical system, perioperative care, peritoneal cytology, physical examination, plain X-ray, polycycstic ovary morphology, polycystic ovary syndrome, polymerase ε, polymerase ε mutated, polymerase ε mutation, polymerase ε testing, polymerase epsilon, polymerase epsilon mutated, polymerase epsilon mutation, positron emission tomography, positron emission tomography-computed tomography, postoperative care, postoperative complications, postoperative recurrence, pregnancy, pregnancy outome, preoperative care, preoperative staging, preoperative work-up, preservation, progesterone, progesterone receptor, progesterone receptor status, progestin, progestin receptor, prognosis, prognostic factor, prognostic value, prophylactic hysterectomy, prophylactic surgery,quality of health care, quality of life, radical hysterectomy, recurrence, recurrent disease, relapse, reoperation, reproductive potential, reproductive surgery, residual disease, residual tumour, response, restaging, risk factors, risk groups, robot-assisted surgery, robotic laparoendoscopic single-site approach, robotic approach, robotic surgery, salpingectomy, salvage surgery, salvage treatment, sensitivity, sentinel lymph node, side effects, sentinel lymph node dissection, sentinel lymph node mapping, specificity, spontaneous abortion, spontaneous pregnancy, staging, staging procedures, standardized uptake value, stillbirth, surgery, surgical management, surgical outcome, surgical outcome criteria, surgical procedures, surgical resection, surveillance, survival, survival rate, survival analysis, synchronous disease, systematic lymphadenectomy, tamoxifen, time to pregnancy, toxicity, transvaginal ultrasound, treatment outcome, tumour, tumour differentiation, tumour characteristics, tumour supressor protein p53, ultra minimally invasive approach, ultra minimally invasive surgery, ultrasonography, unilateral salpingo-oophorectomy,vaginal brachytherapy, vascular endothelial growth factor, weight loss, weight loss interventions, weight reduction, X-ray. |
|  |  |
|  |  |
| Language | English |
|  |  |
|  |  |
| Study design | Priority was given to high-quality systematic reviews and meta-analyses but lower levels of evidence were also evaluated. The search strategy excluded editorials, letters, case reports and *in vitro* studies. The reference list of each identified article was reviewed for other potentially relevant papers. |
|  |  |
|  |  |
