# Supplementary material for: ESGO/ESHRE/ESGE Guidelines for the fertility-sparing treatment of patients with endometrial carcinoma,
Source: Hum Reprod Open. 2023 Feb 6;2023(1):hoac057. doi: 10.1093/hropen/hoac057 (PMC9900425; doi:10.1093/hropen/hoac057)
Supplement: hoac057_Supplementary_Data2 [file hoac057_supplementary_data2.docx]

**Supplementary data 2. LIST OF THE 95 EXTERNAL REVIEWERS**

**Roberto Altamirano**, gynaecological oncology, Chile; **Frederic Amant**, gynaecological oncology, Netherlands; **Barış Ata**, obstetrics & gynaecology, Turkey; **David Atallah**, gynaecological oncology, Lebanon; **Beyhan Ataseven**, gynaecological oncology, Germany; **Manel Barahona Orpinell**, gynaecological oncology, Spain; **Carla Bartosch**, pathology, Portugal; **Sven Becker**, gynaecological oncology, Germany; **Margarida Bernardino**, gynaecological oncology, Portugal; **Nicolò Bizzarri**, gynaecological oncology, Italy; **Marcin Stanislaw Bobinski**, gynaecological oncology, Poland; **Bettina Böttcher**, gynaecological endocrinology/reproductive medicine, Austria; **Margot Bucau**, pathology, France; **Silvia Cabrera**, gynaecological oncology, Spain; **David Cibula**, gynaecological oncology, Czech Republic; **Emma Crosbie**, gynaecological oncology, United Kingdom; **Arianna d'Angelo**, reproductive medicine, United Kingdom; **Nagindra Das**, gynaecological oncology, United Kingdom; **Berta Diaz-Feijoo**, gynaecological oncology, Spain; **Santiago Domingo**, gynaecological oncology, Spain; **Aboubakr Elnashar**, obstetrics & gynaecology, Egypt; **Francesco Fanfani**, gynaecological oncology, Italy; **Mathias Fehr**, gynaecological oncology, Switzerland; **Anis Feki**, obstetrics & gynaecology, Switzerland; **Annamaria Ferrero**, gynaecological oncology, Italy; **Daniela Fischerova**, gynaecological oncology, Czech Republic; **Antónia Furtado**, pathology, Portugal; **Prafull Ghatage**, gynaecological oncology, Canada; **Carolina Gomes**, obstetrics & gynaecology, Portugal; **Clémentine Gonthier**, gynaecological oncology, France; **Mikel Gorostidi**, gynaecological oncology, Spain; **Benedetta Guani**, obstetrics & gynaecology, Switzerland; **Esther Guerra**, pathology, Spain; **Murat Gultekin**, gynaecological oncology, Turkey; **David Hardisson**, pathology, Spain; **Viola Heinzelmann-Schwarz**, gynaecological oncology, Switzerland; **Gines Hernandez-Cortes**, obstetrics & gynaecology, Spain; **Antonio Simone laganà**, obstetrics & gynaecology, Italy; **Joel laufer**, gynaecological oncology, Uruguay; **Ibon Jaunarena**, gynaecological oncology, Spain; **Kirsten Jochumsen**, gynaecological oncology, Denmark; **Ioannis Kalogiannidis**, gynaecological oncology, Greece; **Vesna Kesic**, gynaecological oncology, Serbia; **Gurkan Kiran**, gynaecological oncology, Turkey; **Jaroslav Klát**, gynaecological oncology, Czech Republic; **Martin Koskas**, gynaecological oncology, France; **Gunnar Kristensen**, gynaecological oncology, Norway; **Kim Seng Law**, gynaecological oncology, Taiwan; **Umberto Leone**, gynaecological oncology, Italy; **Louis Ignacio Lete**, obstetrics & gynaecology, Spain; **Lasa Iñaki Lete**, gynaecological oncology, Spain; **Jose Claudio Maanon**, gynaecological oncology, Spain; **Tiziano Maggino**, gynaecological oncology, Italy; **Claudia Mateoiu**, pathology, Sweden; **Patrice Mathevet**, gynaecological oncology, Switzerland; **Mary McCormack**, radiation oncology, United Kingdom; **Miloš Mlynček**, gynaecological oncology, Slovakia; **Philippe Morice**, gynaecological oncology, France; **Esther L Moss**, gynaecological oncology, United Kingdom; **Sabina Murshudova**, gynaecological oncology, Azerbaijan; **Eva Myriokefalitaki**, gynaecological oncology, United Kingdom; **Henrique Nabais**, gynaecological oncology, Portugal; **Gregg Nelson**, gynaecological oncology, Canada; **Eva-Maria Niine-Roolaht**, gynaecological oncology, Estonia; **Dearbhaile O'Donnell**, medical oncology, Ireland; **Felipe Ojeda**, gynaecological oncology, Spain; **Maria Papageorgiou**, patient, Greece; **Vanda Patricio**, gynaecological oncology, Portugal; **Fedro Alessandro Peccatori**, obstetrics & gynaecology, Italy; **Anna Myriam Perrone**, gynaecological oncology, Italy; **Suzana Pessini**, gynaecological oncology, Brazil; **Hanny Pijnenborg**, gynaecological oncology, Netherlands; **Kazimierz Pityński**, gynaecological oncology, Poland; **Mario Preti**, medical oncology, Italy; **Mikuláš Redecha**, gynaecological oncology, Slovakia; **Vera Ribeiro**, obstetrics & gynaecology, Portugal; **Andres Sacristan**, gynaecological oncology, Spain; **Yakir Segev**, gynaecological oncology, Israel; **Aliyev Shamistan**, gynaecological oncology, Azerbaijan; **Tayup Simsek**, gynaecological oncology, Turkey; **Vasileios Sioulas**, gynaecological oncology, Greece; **Smrkolj Spela**, gynaecological oncology, Slovenia; **Erik Soegaard-Andersen**, gynaecological oncology, Denmark; **Artem Stepanyan**, gynaecological oncology, Armenia; **Maciej Stukan**, gynaecological oncology, Poland; **Alina Sturdza**, radiation oncology, Austria; **Germana Tognon**, gynaecological oncology, Italy; **Antonio Travaglino**, pathology, Italy; **Helen Trihia**, pathology, Greece; **Stefano Uccella**, obstetrics & gynaecology, Italy; **Zdravka Veleva**, obstetrics & gynaecology, Finland; **Ana Vilar**, gynaecological oncology, Spain; **Vit Weinberger**, gynaecological oncology, Czech Republic; **Henrica Werner**, gynaecological oncology, Netherlands; **Jacek Jr Wilczynski**, gynaecological oncology, Poland.
